# Recent advances in supramolecular antidotes

**DOI:** 10.7150/thno.53459

**Published:** 2021-01-01

**Authors:** Hang Yin, Xiangjun Zhang, Jianwen Wei, Siyu Lu, David Bardelang, Ruibing Wang

**Affiliations:** 1 State Key Laboratory of Quality Research in Chinese Medicine, Institute of Chinese Medical Sciences, University of Macau, Taipa, Macau, China.; 2 Green Catalysis Center, College of Chemistry, Zhengzhou University, 100 Kexue Road, Zhengzhou 450001, China.; 3 Aix Marseille Univ, CNRS, ICR, Marseille, France.

**Keywords:** Poisons, Macrocycles, Supramolecular Chemistry, Antidotes, Host-guest

## Abstract

Poisons always have fascinated humankind. Initially considered as deleterious or hazardous substances, the modern era has witnessed the controlled utilization of dangerous poisons in medicine and cosmetics. Simultaneously, antidotes have become crucial as reversal agents to counteract the effects of a poison, and they are also used today to positively cancel the benefits of a poison after use. Currently, the majority of poisons are composed of small molecules. This review focuses on recent developments to reverse or prevent toxic effects of poisons by encapsulation in host molecules. Cyclodextrins, cucurbiturils, acyclic cucurbituril derivatives, calixarenes, and pillararenes, have been reported to largely impact the effects of toxic compounds, thus extending the current paradigm of small molecule antidotes by adding a new family of macrocyclic compounds to the current arsenal of antidotes. Along this line of research, endogenous "harmful" species are also sequestered by one or more of these supramolecular host molecules, expanding the potential of supramolecular antidotes to diverse therapeutic areas.

## Introduction

**Figure 1:**
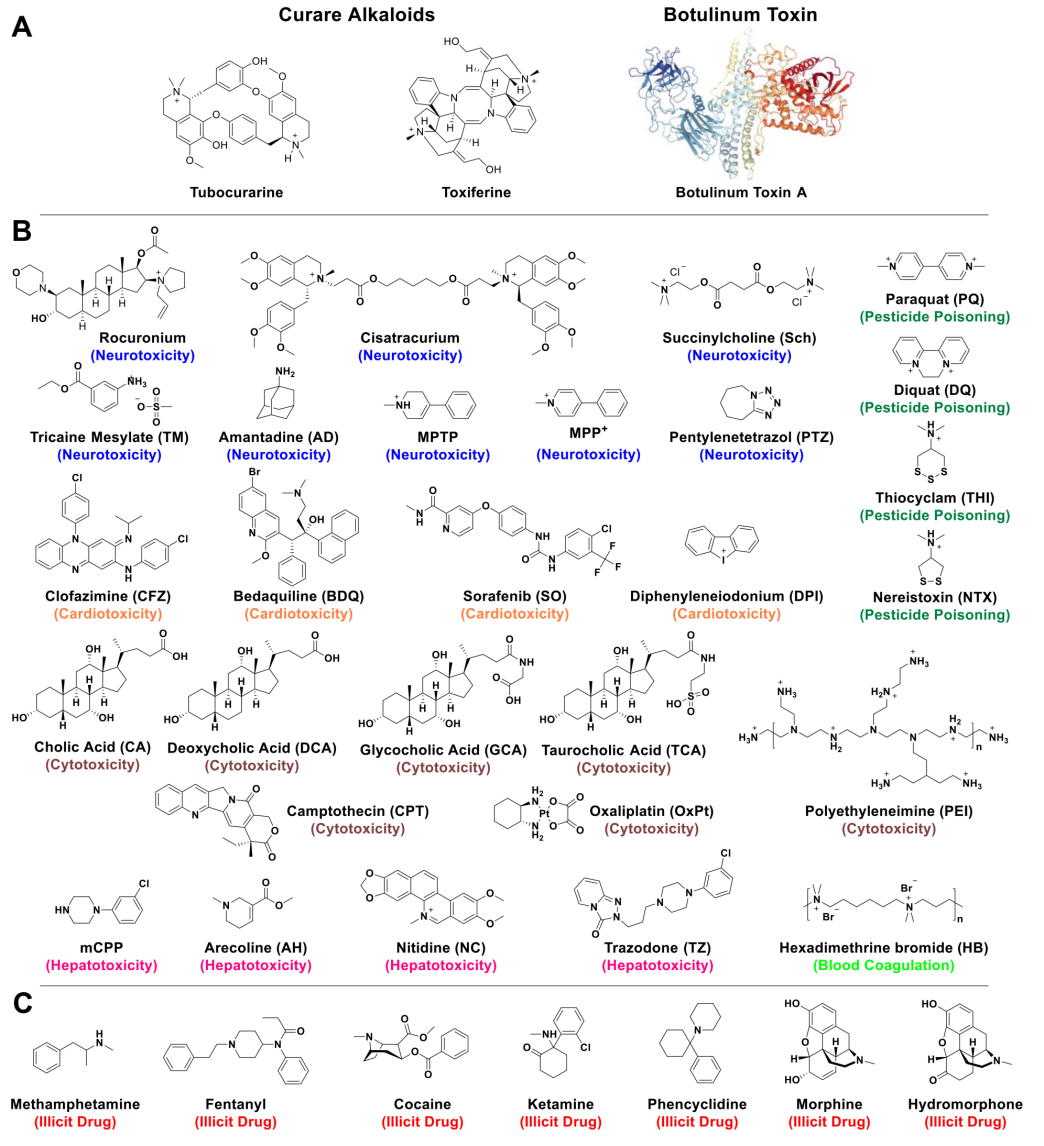
Structures of toxic compounds discussed in this review. Examples of natural toxins (A) present in curares extracted from Amazonian plants or synthesized by bacteria like botulinum toxin. The initial clinical use of curare toxins as muscle relaxants has progressively been replaced by synthetic analogues like Rocuronium or Cisatracurium. Structures of toxic (B) and illicit (C) molecules discussed in this review.

The utilization of poisons (*compounds causing harm, injury or death*) seems to have preceded the invention of writing (beginning of history) by at least 500 years 1. With various applications including probable uses for hunting and social recognition/promotion within early human groups, poisons have then mostly been used for suicide, pest-control, and assassination since Antiquity and generally up to the Renaissance period. According to the World Health Organization, unintentional poisoning remains a problem for modern society, with several million people affected each year 2. Yet, new forms of poisons have started to be widely applied for example as pesticides, key intermediates in the chemical industry, disinfectants, conservatives, or in medicine for instance in surgery (i. e. curare alkaloids 3, previously discovered and used by Amazonian Indians, Figure 1A) or in cosmetics (botulinum toxin 4, a neurotoxic protein that is also the most toxic substance known, Figure 1A). Excluding physical instances, poisons can be chemicals (ions or small molecules) or biologicals (peptides or proteins such as those found in animals' venoms).

In parallel, many antidotes (*substances able to counter the effects of a poison or a disease*) have been discovered with time (such as mithridate, an ancient antidote with complex formulation) and most lethal poisons today have antidotes, such as cyanide (antidote sodium nitrite or thiosulfate), opioids (antidote naloxone), lead (antidote the succimer chelator), heparin (antidote protamine sulfate) or the three medications methotrexate, trimethoprim and pyrimethamine sharing the same antidote: leucovorin. Modern approaches include the production of specific antibodies to counter the effects of venoms 5. However, several poisons still do not have antidotes, such as the alkaloids aconitine (from acotinum plant sometimes used in medicine), or coniine, N-methylconiine and conhydrine (from hemlock plant), famous as the basis for the beverage forced to swallow and having caused the death of the Greek philosopher Socrates (399 BC). The glycoprotein ricin, besides its mention in several fictions, is one of the most toxic substances 6 for which there is no known antidote and that has seriously been considered as a warfare agent. As mentioned above, besides antibodies used as antidotes against venoms, most antidotes are small molecules.

**Figure 2:**
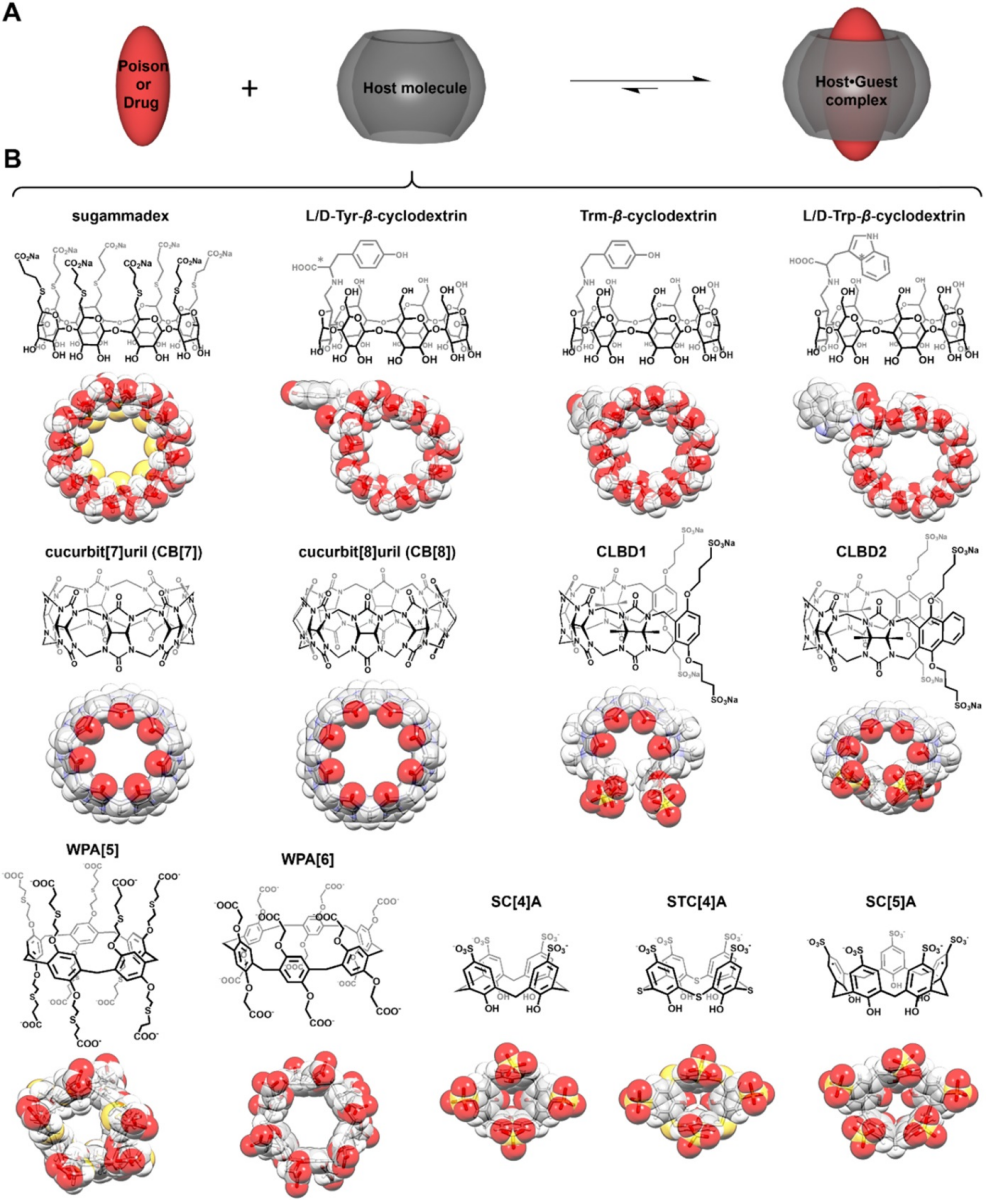
Structures of host molecules used as antidotes. Principle of poison (or drug) capture (A) by synthetic hosts (B). In panel B, cyclodextrins are on the top line, cucurbiturils and acyclic cucurbiturils in the middle one, and pillararenes and calixarenes are at the bottom.

In the past 30 years, the Nobel Prize in Chemistry has been awarded twice to researchers focused on supramolecular systems, more specifically for their contributions toward “*the development and use of molecules with structure-specific interactions of high selectivity*” (1987) and for “*the design and synthesis of molecular machines*” (2016) 7. For both research fields, macrocyclic compounds were central in the findings that have led to major breakthroughs. Yet, despite these significant advances, practical applications stemming from supramolecular chemistry remain relatively limited 8-11. As perhaps one of the most famous families of macrocycles, cyclodextrins (artificial host molecules of natural origin) have started to be used in the pharmaceutical and cosmetic industries (Figure 2) 12-13. In the meantime, supramolecular chemists have developed numerous other host molecules with various topologies, including some resembling crowns (crown ethers), cups (calix[*n*]arenes), pumpkins (cucurbit[*n*]urils), clips (acyclic cucurbit[*n*]uril congeners), or tubes (pillar[*n*]arenes, see Figure 2), *n* denoting the number of monomer repeat units, among others 14.

Indeed, supramolecular systems have achieved preliminary success for biomedical applications 15-16. For instance, some supramolecular polymers even reached the clinic 17-18. Among a variety of supramolecular systems, macrocycles and related host molecules have been widely studied in biomedical applications due to their stable physical/chemical properties, batch-to-batch consistency, and relatively high biocompatibility 19. Scientists have discovered that simple host-guest complexes can make good drug delivery systems, with marked advantages for some of them to alleviate or reverse side-effects or toxicities of drugs both *in vitro* and *in vivo* when these drugs are encapsulated by host molecules, thanks to their relatively strong host-guest interactions mediated via hydrogen-bonding, electrostatic interaction, and/or hydrophobic interactions 19-21. Indeed, the toxicity or unforeseen side-effects of biologically active molecules can limit their clinical applicability or cause the interruption of a drug development program. With the rapid growth of the number of reported host molecules 22-24, many macrocycles have shown interesting binding properties toward bioactive compounds, and some of them, good inhibition/reversal effects against toxic compounds and drugs exhibiting adverse side-effects, although there has been only one commercial success (Sugammadex) in the development of host molecules as clinically approved antidotes 20-21, 25. However, the potential of host molecules as promising antidotes that have been extensively investigated *in vitro* and *in vivo* has never been summarized. In this context, host molecules having shown inhibition or reversal of biological properties for a given compound are the objects of the present review with highlights on the most promising host molecules used as antidotes, and on products that are now on the market.

## Cyclodextrins

Cyclodextrins (CDs, Figure 2B) are considered as one of the oldest known families of host molecules, and their discovery over a century ago predates the establishment of supramolecular chemistry as a scientific discipline. CDs are sugar-based cyclic oligomers made from the bacterial degradation of starch and have a versatile hydrophobic cavity amenable for binding a plethora of guest molecules 26. The excellent biocompatibility and the ability to enhance solubility of insoluble drugs via host-guest interactions have made CDs as one of the most widely applied excipients in pharmaceutics 27. However, chemically functionalized CDs may bind strongly with toxic species, thereby exhibiting reversal effects.

### Reversal of neuromuscular blocking agents

**Figure 3:**
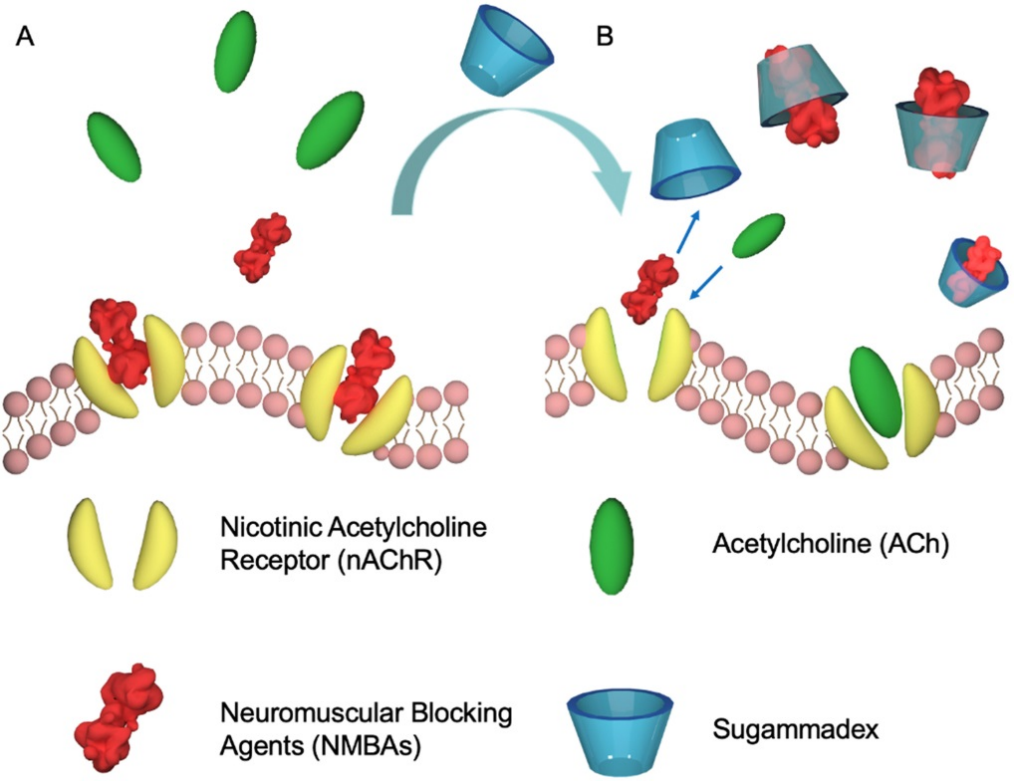
Proposed mechanism for the recovery of natural partners of nAChR membrane proteins by sugammadex. Schematic representation of the mechanism of sugammadex as a supramolecular antidote to reverse NMBAs-induced neuromuscular blocking effects by competitive binding.

Even if CDs are usually considered as non-toxic, they did not attract much attention as an antidote until Zhang and co-workers used a γ-CD derivative (sugammadex, Figure 2B) to reverse the effects of neuromuscular blocking agents (NMBAs) in 2002 (Figure 3) 28-29.

NMBAs are mono- and most often di-quaternary ammonium compounds derived from curare or malouetine (like rocuronium or cisatracurium, Figure 1), binding post-synaptic acetylcholine receptors and causing paralysis of skeletal muscles 30. They are often used to complement anesthesia. With a high binding affinity *K*_a_ of (1.05±0.16)×10^7^ M^-1^ between sugammadex and rocuronium, the host compound could efficiently reverse the rocuronium-induced neuromuscular blocking effect *in vivo*, that has paved the way to the development of supramolecular antidotes 28. The success of this approach was soon followed by subsequent studies, further establishing sugammadex as an effective antidote 31. Finally, sugammadex, with the trade name of Bridion®, has been developed and commercialized by Organon, which was acquired by Schering-Plough and subsequently merged with Merck. Despite this success, sugammadex is not without shortcomings. Its approval by the U.S. Food and Drug Administration (FDA) has been delayed due to its association with anaphylaxis 32. In addition, sugammadex-induced bradycardia and asystole cannot be ignored 33. These side-effects could be due to unspecific binding and hence, new host molecules with better affinities would be interesting to test.

### Clearance of bile acids

Liu and co-workers studied the binding of bile acids (BAs) such as cholic acid (CA), deoxycholic acid (DCA), glycocholic acid (GCA), and taurocholic acid (TCA, Figure 1B), with L/D-tyrosine-modified β-CDs (L/D-Tyr-β-CDs) or L/D-tryptophan-modified β-CDs (L/D-Trp-β-CDs, Figure 2B) and strong affinities were observed in most cases 34. However, Liu et al. postulated that the -COOH groups on both host and guest molecules could have hindered an efficient recognition between these β-CD derivatives and BAs due to electrostatic repulsions between carboxylate groups. They thus synthesized a tyramine-modified β-CD (Trm-β-CD) to verify their assumption 35. In general, this new host has shown stronger affinities than L/D-Tyr-β-CDs and L/D-Trp-β-CDs toward BAs. Both *in vitro* (HT-29 and HCT-116 cell lines) and *in vivo* (mice) studies showed that Trm-β-CD can reverse the cytotoxicity of DCA and accelerate the clearance of blood DCA, suggesting a promising future for this host with respect to intrahepatic cholestasis and other BA-related diseases.

## Calixarenes

Calix[*n*]arenes (C[*n*]As) were named like this on account of their shape, resembling that of a chalice or grail (Figure 2B) 36. These host molecules have been studied for decades and many accomplishments have been achieved in various research areas 14. However, the poor water solubility and relatively high toxicity of these compounds have limited their application in biomedical sciences 37. Despite these drawbacks, scientists have developed several methods to facilitate the use of C[*n*]As in the biomedical field. The most promising strategy turned to be the synthesis of water soluble and less toxic C[*n*]A derivatives: *p*-sulfonatocalix[*n*]arenes (SC[*n*]As, *n* = 4-8, Figure 2B) 37.

### Antidote for pesticide poisoning

**Figure 4:**
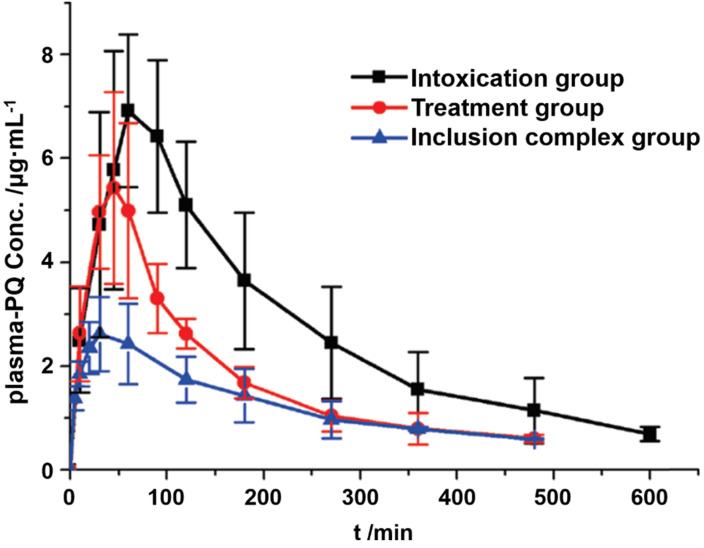
Plasma concentration of PQ against time after oral administrations of PQ, inclusion complex, and treatment with C[4]AS 30 min after PQ poisoning. Reprinted from ref. 39. Copyright 2011 American Chemical Society.

Liu and co-authors have studied the recognition of the pesticide paraquat (PQ, Figure 1B) by SC[*n*]A (*n* = 4, 5) in several phosphate buffers (pH = 2.0, 7.2, and 12.0) 38. PQ forms a stable complex with SC[4]A (binding constant *K*_a_ ≈ 10^4^ M^-1^ at all pH), while the corresponding value for the PQ•SC[5]A complex (*K*_a_ ≈ 10^3^ M^-1^) raised to 10^5^ M^-1^ when the pH was increased from 2.0 to 12.0 38. Later, the potential of SC[4]A, SC[5]A, and a newly introduced *p*-sulfonatothiacalix[4]arene (STC[4]A) was studied as host compounds for the treatment of viologens poisoning using paraquat and diquat as poisons 20. Upon encapsulation, the transformation of oxygen to superoxide (O_2_^●―^) was reduced, thereby inhibiting the generation of hydroxyl radicals (HO^●^), and decreasing the toxicity of viologens. Subsequently, the PQ•SC[4]A complex was selected for an *in vivo* pharmacokinetic study involving oral administration 39. Compared to the group of rats treated with PQ alone, the group treated with PQ•SC[4]A showed lower PQ concentrations in plasma (Figure 4).

These results were supported and tentatively explained by an *in vitro* intestinal absorption study, that showed an inhibition of PQ absorption by the intestines, when SC[4]A was present 39. SC[*n*]A macrocycles are further chemically tunable and could thus be another family of supramolecular antidotes in the near future.

### Antidote of heparin

Unfractionated heparin (UFH) is widely used in clinical practice as an anticoagulant agent. However, when overdosed or administered in sensitive populations, UFH may cause several side effects including excessive bleeding 40. The only clinically available UFH neutralization agent, protamine, has several risks as well associated with its use, including allergic reactions in a large sub-population of patients 40. Hence, it is essential to develop novel UFH neutralizers to reverse heparinization in an effective and safe manner, as a replacement or supplementary of protamine. Very recently, a biocompatible oligoethylene glycol functionalized guanidinecalixarene (GC[4]AOEG), which binds with UFH with a high binding affinity (10^7^ M^-1^), was designed as a supramolecular antidote against UFH. *In vivo* tests in three mouse bleeding models showed that GC[4]AOEG can effectively alleviate excessive bleeding induced by UFH with an excellent safety profile, indicating a great potential of GC[4]AOEG for clinical translation as a UFH neutralizer 40.

## Cucurbiturils

Cucurbit[6]uril had received only modest attention until the groups of Kim and Day successfully isolated several homologues (cucurbit[*n*]urils: CB[*n*]s, *n* = 5-8, Figure 2B) forming in 2000 a new family of host molecules and opening new avenues for macrocyclic chemistry 41-51. Like CDs, these synthetic macrocycles are usually considered as relatively non-toxic compounds 52-55. In 2004 56 and 2005 57, two seminal papers have reported the encapsulation of platinum drugs in CB^7^ as an approach to reduce their toxicity. In 2011, Gilson and co-authors found that CB^7^ could tightly bind amantadine (AD, Figure 1B, an antiviral medication also used against Parkinson's disease; *K*_a_ = 1.7±0.8×10^14^ M^-1^) 58. This discovery demonstrated that CB^7^ could be employed as a latent competitor against natural receptors for the binding of small molecules. In parallel, the Wang's group has systemically studied a series of cases for which CB^7^ was used as a potential antidote or toxicity-inhibitory agent. In particular, CB^7^ was found to have a significant potential as a neural toxicity reversal agent 59-61.

### Alleviation of neurotoxicity

**Figure 5:**
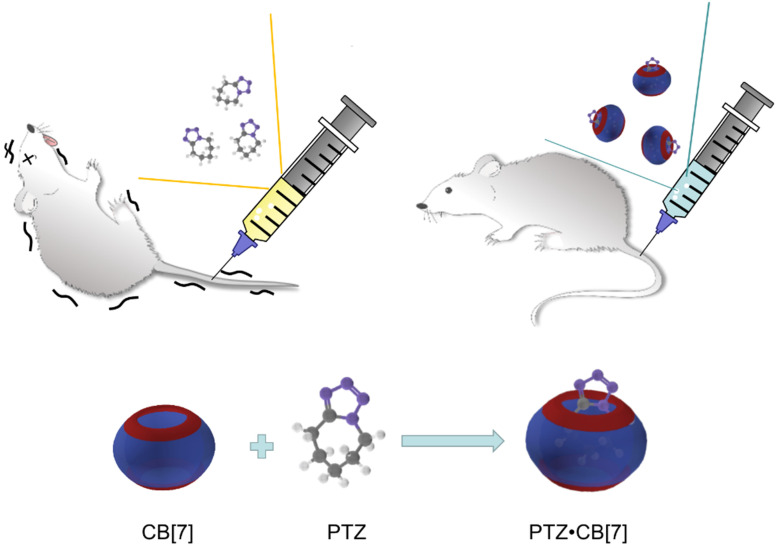
Scheme showing the reduction of neurotoxicity of PTZ administered with CB^7^ in mice.

CB^7^ was found to accelerate the recovery of zebrafish that had been anaesthetized by tricaine mesylate (TM, Figure 1B), an FDA-approved general anesthetic 59. In addition, CB^7^ was found to inhibit the neurotoxicities of *N*-methyl-4-phenyl-1,2,3,6-tetrahydropyridine (MPTP, Figure 1B), a neurotoxin that is used to construct Parkinson's disease models, and its active metabolite *N*-methyl-4-phenylpyridine (MPP^+^) in a study relying on zebrafish 60. CB^7^ was then shown to reduce the toxicity of pentylenetetrazol (PTZ, Figure 1B), a neurotoxin inducing seizure, on zebrafish and mice (Figure 5) 61.

Results obtained with these models are presumably due to the relatively strong binding affinities between CB^7^ and the neurotoxic compounds. Indeed, CB^7^ forms 1:1 host-guest complexes with TM, MPTP, MPP^+^, and PTZ, with relatively high binding constants of the order of ~10^5^ M^-1^, starting to make CB^7^ a good competitor impeding the binding between neurotoxins and their natural receptors 59-61. Following the reversal of neurotoxicities by CB^7^, the ability of this macrocycle to reverse or inhibit other toxicities has been evaluated.

### Alleviation of cardiotoxicity

Cardiotoxicity is one of the high-risk side effects of bioactive compounds, often inducing cardiac dysfunctions. In this context, CB[*n*]s pleasingly showed good efficacy in alleviating cardiotoxicities of several bioactive molecules 62-65. For instance, clofazimine (CFZ, Figure 1B) is a drug that was developed for the treatment of tuberculosis but suffers from cardiotoxicity and poor solubility in water 62. After encapsulation in CB^7^ (*K*_a_ ≈ 10^4^~10^5^ M^-1^), the water solubility of CFZ increased in both acidic and neutral media, and *in vitro* and *in vivo* tests have shown drops of CFZ cardiotoxicity without affecting its antimycobacterial activity 62. Bedaquiline (BDQ, Figure 1B), another anti-tuberculosis drug, is also prone to the same issues. The solubility of BDQ increased with CB^7^ by a factor of 0.27-fold of the concentration of CB^7^. Meanwhile, *in vivo* tests including a set of physiological parameters for cardiac functions showed that the cardiotoxicity of BDQ was dramatically decreased in the presence of CB^7^ ^63^. Sorafenib (SO, Figure 1B) is a small-molecule kinase inhibitor (SMKI) that is widely used in the treatment of various cancers 64, but is also characterized by a significant cardiotoxicity. Relatively high affinity between SO and CB^7^ (*K*_a_ = (2.87 ± 0.13)×10^5^ M^-1^) enabled this host to decrease SO cardiotoxicity without affecting the desired activity, as confirmed *in vitro* (with SMMC7721 cell lines) and *in vivo* (with zebrafish models) 64. Diphenyleneiodonium (DPI, Figure 1B) is an uncompetitive inhibitor of flavoenzymes able to reduce the activity of NADPH oxidase, xanthine oxidase, and nitric oxide synthase 65. However, a latent cardiotoxicity of DPI presents risks when used *in vivo*. Both CB^7^ and CB^8^ form stable complexes with DPI in aqueous solution, CB^7^ being singly complexed and CB^8^ doubly bound (*K*_a DPI•CB7_ = (3.13±0.16)×10^4^ M^-1^, and *K*_a DPI•CB8_ = 2.26×10^12^ M^-2^), but CB^7^ showed better performance in reducing the cardiotoxicity of DPI, likely due to its better solubility compared to that of CB^8^ ^65^.

### Alleviation of hepatotoxicity

Natural products are an important source of new medicines, but their hepatotoxicities can present serious concerns. For instance, arecoline (AH, Figure 1B) is an active compound extracted from Areca nut that has been studied for the treatment of several neurological disorders, but also relatively to the cardiovascular and digestive systems. However, AH can show severe hepatotoxic effects 66 so CB^7^ was tested as a supramolecular antidote to minimize its hepatotoxicity. The association between AH and CB^7^ was first studied and has shown formation of a 1:1 complex with a binding affinity *K*_a_ of (1.21±0.12)×10^3^ M^-1^. Subsequent work demonstrated a reduction of the hepatic toxicity of AH *in vitro* based on tests performed on L02 cell lines 66. Nitidine chloride (NC, Figure 1B) is a natural alkaloid extracted from the root of *Zanthoxylum nitidum* 67 and a promising drug candidate against cancer, but further studies were impeded by its hepatic toxicity. A novel formulation of NC with CB^7^ was tested, enabling to boost its anti-cancer activity (IC_50_ = 2.94±0.15 μM compared to 7.28±0.36 μM for free NC) 67 while reducing its hepatotoxicity (IC_50_ = 3.48±0.49 μM), by a factor of ~2 (IC_50_ = 6.87±0.80 μM for free NC) *in vitro*. Trazodone (TZ, Figure 1B) is an FDA-approved drug that has been developed for the treatment of depression. However, its active metabolite, *m*-chlorophenylpiperazine (mCPP), has been associated with hepatotoxicity. An *in vitro* study showed that CB^7^ could complex these two compounds and significantly decrease the hepatotoxicities caused by TZ and mCPP 68. These results were confirmed *in vivo* highlighting the potential of this formulation for clinical applications 68.

### Alleviation of general cytotoxicity

The administration of anti-cancer drugs is often accompanied by unexpected side-effects induced by their cytotoxicity. Camptothecin (CPT, Figure 1B) is a potent anti-cancer agent used to treat various cancers 69 but it can be converted into an active lactone form and a toxic carboxylate analogue, thereby limiting its applicability. Studies have shown that the structural changes of CPT can be limited by formation of supramolecular complexes with CB^7^. Both *in vitro* and *in vivo* investigations have shown that the presence of CB^7^ could alleviate the non-specific toxicity of CPT 69. Zhang and co-authors have studied a supramolecular chemotherapy based on the formation of complexes between the anti-cancer drug oxaliplatin (OxPt, Figure 1B) and CB^7^, ^57^, ^70^. Their study has demonstrated that the presence of CB^7^ could significantly reduce the cytotoxicity of OxPt *in vitro* (on healthy colorectal NCM460 cells), and improve its anti-cancer activity by competitive binding with spermine which is overexpressed in some cancer cells such as HCT116 or HT-29 cells.

Besides common drugs, the toxicity of polymers can also be a serious source of concern. Polyethylenimine (PEI, Figure 1B) has been studied as a gene delivery vehicle 71. However, the applicability of high molecular weight PEI (branched, 25 kDa) that have shown better gene delivery, is limited by their non-specific cytotoxicity. In this context, CB^7^ was shown to significantly decrease the cytotoxicity of PEI most likely by multiple host binding 71. Likewise, the supramolecular shielding strategy works also well with hexadimethrine bromide (HB, or polybrene Figure 1), a polycation that can neutralize heparin and control internal bleeding. However, its use can induce severe cases of blood coagulation raising major concerns for the lives of patients 72. CB^7^ can complex each repeating unit of HB with a binding affinity *K*_a_ of (1.04±0.19)×10^7^ M^-1^. Both *in vitro* and *in vivo* tests showed that coagulation effects induced by HB can be significantly decreased by this supramolecular encapsulation strategy 72.

### Antidote for pesticide poisoning

**Figure 6:**
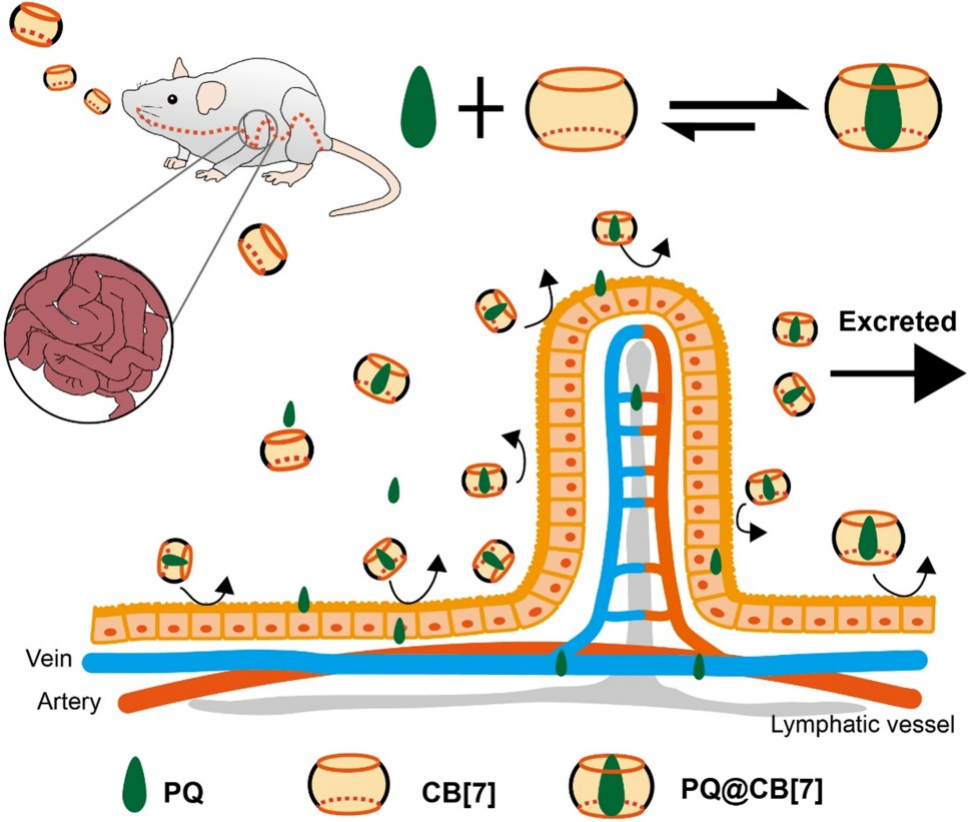
Proposed function of CB^7^ as treatment in PQ detoxification. CB^7^ is orally administered after PQ ingestion. In the stomach or intestine, PQ is trapped by CB^7^, preventing further damages to the intestines and reducing the absorption and tissue distribution of PQ. Most of the pesticide will be excreted as PQ•CB^7^ complexes. Reprinted from ref. 74. Copyright 2019 Ivyspring.

The extensive utilization of pesticides in agriculture is a significant source of pollution and can cause severe harm to ecosystems or humans. For instance, the pesticide nereistoxin (NTX, Figure 1B) and its derivative thiocyclam (THI, Figure 1B) have shown non-selective teratogenic toxicities, tested for alleviation by formation of supramolecular complexes with CB^7^ ^73^. Relatively high binding constants of (1.4±0.15)×10^5^ M^-1^ and (7.46±0.10)×10^5^ M^-1^ for NTX and THI respectively, enabled assessing antidotal effects. *In vivo* tests performed on zebrafish embryos and larvae showed that CB^7^ significantly constrained the teratogenicity of NTX and THI 73. Another example is that of the toxic and famous pesticide paraquat (PQ), still responsible for serious troubles in public health 74. Recently, the Wang's group has studied the antidotal effect of CB^7^ on PQ toxicity both *in vitro* and *in vivo* (Figure 6) 74. While a reduction of PQ's toxicity was noted previously in studies focused on PQ•CB^7^ complexes as anti-cancer agents (*K*_a_ ~ 10^5^ M^-1^) 75, the Wang's group developed a new method to reverse PQ poisoning 74. This work showed that CB^7^ could significantly decrease PQ levels in plasma and major organs, and alleviate their adverse effects via oral administration. In addition, oral administration of CB^7^ within 2 hours post-PQ ingestion could improve the survival rates of mice and extend their survival time, outperforming activated charcoal that is usually used for the treatment of PQ poisoning 74. Finally, with the antidotal effect of CB^7^, the PQ•CB^7^ complex can also potentially be used as a safer herbicide 76.

## Acyclic cucurbiturils

One of the key advantages of CB[*n*]s is their rigid structures, but this can also limit their use in certain cases. Isaacs and co-authors have developed a series of flexible, clip-like CB[*n*]s known as acyclic cucurbit[*n*]uril-type congeners (acyclic CB[*n*]) 77. Calabadion 1 and calabadion 2 (abbreviated CLBD1 and CLBD2, respectively, see Figure 2B) have a flexible C-shape structure that can grasp many guest molecules in water. These acyclic CB[*n*]s have shown good biocompatibility and strong binding affinities toward a wide range of molecules 25, 77-78, including NMBAs such as rocuronium and cisatracurium 25, 79-80.

### Reversal of neuromuscular blocking agents

*In vivo* studies have revealed that CLBD1 could accelerate the recovery of spontaneous breathing, and reduce the time required to reach a train-to-four ratio of 0.9 for rats (given rocuronium and cisatracurium), from minutes to seconds, outperforming the recommended neostigmine/glycopyrrolate treatment 79. On the other hand, rocuronium was shown to be 89-fold better complexed by CLBD2 (*K*_a_ = 3.4×10^9^ M^-1^) than by sugammadex (*K*_a_ = 3.8×10^7^ M^-1^). Accordingly, CLBD2 provided a better reversal effect than sugammadex *in vivo* 80.

### Reversal of toxicity of illicit drugs

**Figure 7:**
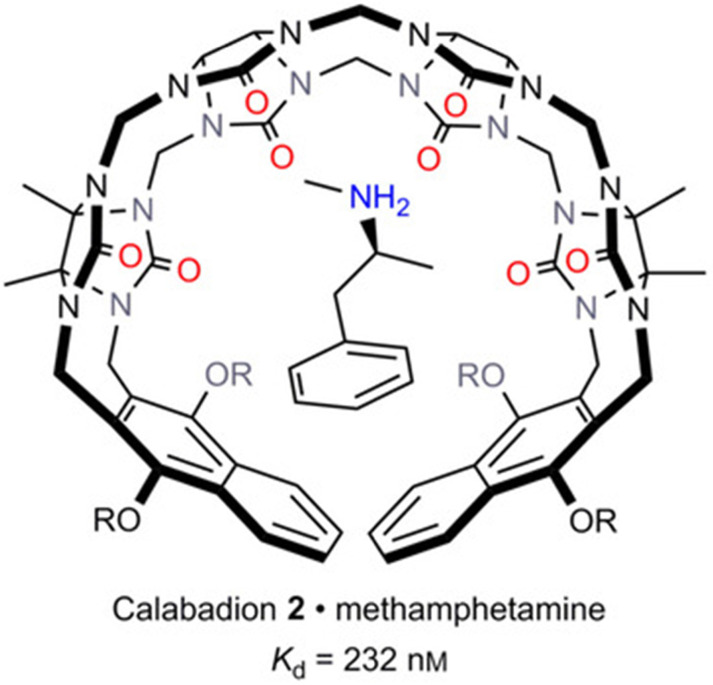
Capture of methamphetamine by CLBD2. Reprinted from ref. 81. Copyright 2017 John Wiley & Sons, Inc.

More recently, Isaacs and co-authors further explored the potential of acyclic CB[*n*] derivatives as supramolecular antidotes. The complexation of various illicit drugs (methamphetamine, fentanyl, cocaine, ketamine, phencyclidine, morphine and hydromorphone, Figure 1B) by CLBD1, CLBD2, and other host molecules (CB^7^, C[4]AS and HP-*β*-CD) was investigated. Among all hosts, CLBD2 showed a good binding affinity for methamphetamine (*K*_a_ = (4.3±1.0)×10^6^ M^-1^, Figure 7), enabling to reverse the hyperlocomotive activity of rats treated with methamphetamine 81.

## Pillararenes

As one of the latest series of host molecules discovered, pillar[*n*]arenes (PA[*n*]s) have experienced a rapid development during the past decade 82-85. Many applications of PA[*n*]s have been reported. However, as is the case for most host molecules, water solubility was a key barrier impeding their biological applications. In 2012, Huang and co-workers prepared a water soluble pillar[6]arene (WPA^6^, Figure 2B) with an excellent potential for biological applications 86. The strong binding affinity of WPA^6^ for PQ (*K*_a_ = (1.02±0.10)×10^8^ M^-1^) suggested that WPA^6^ could be a potent antidote 87.

### Alleviation of cytotoxicity

This macrocycle was so used in a supramolecular chemotherapy approach to decrease the cytotoxicity of OxPt while improving its anti-cancer activity. The good binding affinity between WPA^6^ and OxPt in phosphate buffer at pH 7.4 and 37.0 °C enabled to decrease the cytotoxicity of OxPt 88-89.

### Reversal of neuromuscular blocking agents

Succinylcholine (Sch, Figure 1) is the only depolarizing NMBA that is widely used for rapid sequence induction in emergency rooms 90. However, its use can be accompanied by severe side-effects, such as hyperkalemia and cardiac arrest. Sch can be complexed by SC[4]A, CB^7^ and WPA^6^ *in vitro* with respective association constants *K*_a Sch•C[4]AS_ ~ 10^4^ M^-1^, *K*_a Sch•CB7_ ~ 10^6^ M^-1^ and *K*_a Sch•WPA6_ ≈ 3.4×10^6^ M^-1^. Among these hosts, WPA^6^ exhibited excellent antidotal effects. *In vivo* studies showed that the encapsulation of Sch by WPA^6^ reduced the guest side-effects', such as the incidence of cardiac arrhythmia, high serum potassium levels, and muscular damage 90. Inspired by the success of sugammadex, Stoikov and co-workers synthesized a water soluble PA^5^, namely WPA^5^ (Figure 2B), containing similar side-chains as those of sugammadex, and aimed at achieving a better reversal effect for the use of rocuronium bromide. Although WPA^5^ required a longer time than enabled by sugammadex for the recovery of muscle contraction after restriction by rocuronium, it still showed a great potential as a new kind of antidote 91. The main properties of macrocycles as antidotes are summarized in Table 1.

## Supramolecular therapeutics via sequestration of endogenous species

All of these above-discussed examples are about sequestration of exogenous toxic/harmful species and counteracting their toxicities via supramolecular encapsulation. Indeed, very often some diseases are induced by, or are featured with, a high concentration of harmful endogenous species or biomarkers, and elimination of these species may inhibit the disease progression. Thus, along the line of “supramolecular antidote” research, efficient sequestration or “trap” of disease-related endogenous species may provide a promising therapeutic strategy for various diseases.

### Supramolecular “trap” of spermine for cancer treatment

Polyamines, including spermine (SPM), spermidine (SPD), and putrescine (PUT), exist in a wide range of living organisms and are essential for cell proliferation and differentiation. Therefore, elimination of free polyamines in cancer cells has become a potential approach to induce cancer cell apoptosis to improve cancer treatment. For instance, Zhang et al. have designed a series of macrocycles-based drug delivery systems, where chemotherapeutic agents get released via competitive binding of SPM to the host molecules 70, 75, 88, 92. The first two CB^7^-based supramolecular drug delivery systems, PQ-CB^7^ ^75^ and OxPt-CB^7^ ^70^, showed enhanced anti-cancer activity due to the synergetic effect of controlled drug release and capture of SPM. Subsequently, a CB^7^ based polyethylene glycol (PEG) was designed as a platform for drug delivery aiming at long systemic circulation while preserving all the benefits of the previously reported supramolecular drug delivery systems 92. Additionally, WPA^6^ was investigated as a supramolecular drug carrier, similar to CB^7^ ^88^. The OxPt-WPA^6^ drug delivery system, with a large difference between the binding affinities of SPM-WAP^6^ and OxPt-WPA^6^, showed much better anti-cancer activity than those based on CB^7^. Very recently, Li et al. synthesized a peptide-PA^5^ conjugate (denoted as P1PA^5^, where P1 refers to RGDSK(N_3_)EEEE) as a supramolecular trap for efficient elimination of free polyamines in cancer cells 93. P1PA^5^ exhibited high binding affinities toward a wide range of polyamines (*K*_a_ of (3.98±0.68)×10^5^ M^-1^, (1.45±0.14)×10^5^ M^-1^, and (2.32±0.68)×10^6^ M^-1^ for SPM, SPD, and PUT, respectively). The efficient, specific cell penetration of the host molecule and the subsequent “trap” of free polyamines in cells by P1PA^5^ yielded good anti-cancer activities both *in vitro* and *in vivo*.

### Supramolecular sequestration of cholesterol for treatment of atherosclerosis and NPC disease

Cholesterol plays a vital role in the progression of atherosclerosis (among several other diseases), since cholesterol is a major component of atherosclerotic plagues and its accumulation and deposition triggers a complex inflammatory response to further promote atherosclerosis. 2-Hydroxypropyl-β-cyclodextrin (HP-β-CD), an FDA-approved excipient to enhance the solubility of numerous lipophilic agents, was found to enhance the solubility of cholesterol 94. In fact, HP-β-CD not only increases the solubility of cholesterol to reduce the formation of cholesterol crystals, but also enhances the production of water-soluble oxysterol to finally realize the anti-inflammatory effects after a complex intracellular metabolism process. As a result, HP-β-CD showed good therapeutic effects toward atherosclerosis *in vivo* 94. However, the poor pharmacokinetics and ototoxicity of HP-β-CD still limit its clinical translation for atherosclerosis treatment. Recently, a β-CD based polymer (β-CDP) was designed to overcome these disadvantages of HP-β-CD, by showing a safer profile and better atherosclerosis treatment effects than HP-β-CD, ascribed to the enhanced pharmacokinetics *in vivo* 95.

In general, there are two metabolism pathways of cholesterol after cellular uptake: 1) transportation from lysosome to endoplasmic reticulum with the assistance of Niemann-Pick type C1/C2 (NPC1/NPC2) transporter, and transformed into cholesteryl esters; 2) metabolized into water-soluble oxysterols 94. The mutations of NPC1 or NPC2 may induce the accumulation of cholesterol in lysosomes and further influence subsequent biological processes, which is known as Niemann-Pick disease type C (often known as NPC disease). NPC causes hepatosplenomegaly, neonatal cholestatic jaundice, splenomegaly, and even death 96. With the same action mechanism to elimination of cholesterol for atherosclerosis treatment 94, HP-β-CD has emerged as a potential agent for the treatment of NPC. Both *in vitro* 97, and *in vivo* studies (including clinical trials) 96 have demonstrated the efficacy of HP-β-CD for NPC treatment with a decent safety profile, providing the only therapeutic hope for NPC patients, and thus far only supramolecular medicine shows the charm.

## Conclusion, Challenges and Perspectives

**Figure 8:**
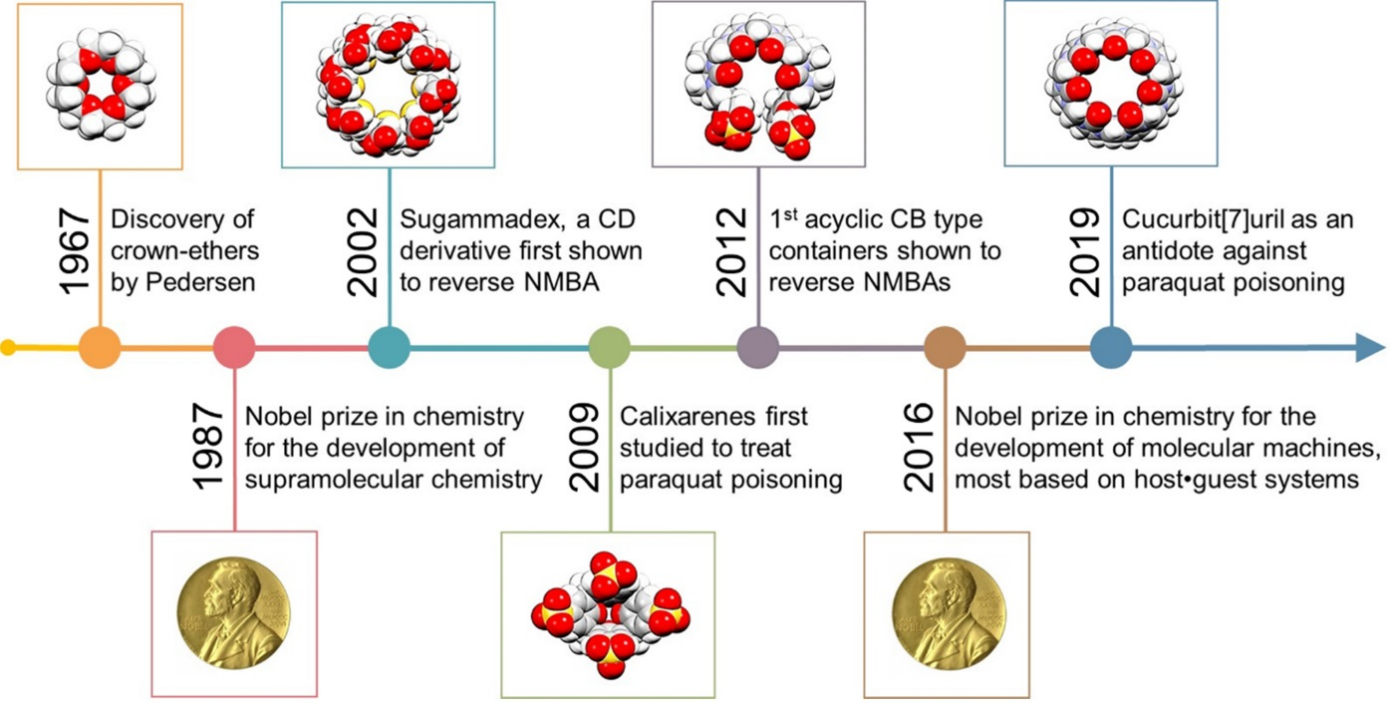
Timeline highlighting selected key developments in supramolecular chemistry and prime macrocyclic antidotes in the fight against poisons.

In summary, we believe that the macrocyclic chemistry has entered a gold era. The number of host compounds reported has never been so high and researchers are now strongly interested in testing rapidly new macrocycles for an also ever-increasing number of applications. Starting with sugammadex, which is the initial host that has been developed to reverse the effects of NMBAs (Figure 8), other host molecules have started to be studied for their inhibition/reversal effects toward bioactive compounds.

As the sole clinically and commercially successful case and as a supramolecular antidote, sugammadex still presents significant risks that have limited its extensive applications. But many host molecules such as cyclodextrins, pillararenes, cucurbiturils, and acyclic cucurbituril derivatives have shown a real potential for the next generation supramolecular antidotes, they can additionally be functionalized to improve affinity and selectivity, and new host molecules are periodically described 14, 24. In the definition given for “*Antidotes*” by the Encyclopedia Britannica, it is mentioned that such a substance can be able to: “*keep … (a poison) … from fitting a receptor at its site of action; or binding to a receptor to prevent the poison's binding there, blocking its action*”. The definition implies that binding affinity and selectivity are key factors that influence the antidotal effects of any given host molecules. A high binding affinity and selectivity would likely afford an effective antidote toward a specific guest species; however, the general applicability would be compromised by the high selectivity, making such an antidote of less "commercial" interest. A low-selectivity host may offer broad-spectrum antidote against a variety of toxic guests, but this often comes with a relatively low efficacy and likely more side effects. These factors have to be considered and balanced in future development of supramolecular antidotes.

Besides the toxicities induced by exogenous species, harmful endogenous species (broadly defined as internal “toxins”/”poisons” once the local concentration is high) may also be sequestered via supramolecular encapsulation for various therapeutic purposes. Thus far, supramolecular medicine has exhibited significant potential for the treatment of cancer, atherosclerosis and NPC disease *in vivo* and even in humans. The unique role of supramolecular host molecules in this case provides a new strategy to treat various diseases that are otherwise difficult to tackle. Under the same principles, diseases caused by other endogenous, harmful substances, such as uric acid, bile acid, high blood sugar, etc. may find practical solutions with delicately designed host molecules that can specifically “trap” these molecules *in vivo*. The field of supramolecular medicine will certainly become burgeoning by then.

Furthermore, with the emergence of protein binding by macrocycles such as calixarenes or cucurbiturils 98-102 there is only one step to imagine macrocycles able to target proteins expressed on the surface of viruses, thereby reducing virus binding and impeding early stages of viral infection, something highly topical after the emergence of the pandemic SARS-CoV-2 virus. There still may be a long way to reach that purpose, but macrocycles and analogous host compounds have already opened fascinating perspectives as supramolecular antidotes in a broader context.

## Figures and Tables

**Table 1:** Effects of supramolecular antidotes toward toxic compounds

Molecule	Toxicity	Antidote	Effect of Antidote	Binding Affinity [Solvent/pH/Temperature (°C)]	Ref.
Rocuronium	Neurotoxicity	Sugammadex	Accelerate recovery from anaesthesia	(1.05±0.16)×10^7^ M^-1^ [50 mM PBS/7.0/25]	28
		CLBD1	Accelerate recovery from anaesthesia	(8.4±0.9)×10^6^ M^-1^ [N.A./N.A./N.A.]	79
		CLBD2	Accelerate recovery from anaesthesia	3.4×10^9^ M^-1^ [N.A./7.4/25]	80
		WPA^5^	Accelerate recovery from anaesthesia	4.5×10^3^ M^-1^ [H_2_O/N.A./19.85]	91
DCA	Cytotoxicity	Trm-β-CD	Decrease the cytotoxicity and accelerate the clearance of blood DCA	(1.57±0.07) ×10^4^ M^-1^ [3% DMSO-PBS/N.A./25]	35
PQ	Pesticide Poisoning	C[4]AS	Alleviate poisoning	~10^4^ M^-1^ [PBS/N.A./25]	39
		C[5]AS	Alleviate poisoning	~10^3^ M^-1^ to 10^5^ M^-1^ [PBS/2.0-12.0/25]	20
		CB^7^	Alleviate poisoning	~10^5^ M^-1^ [HCl-PBS/1.2-7.4/25]	74
		WPA^6^	Alleviate cytotoxicity	(1.02±0.10)×10^8^ M^-1^ [H_2_O/N.A./R.T.]	87
UFH	Complex side effects	GC[4]AOEG	Reverse heparinization	(1.25±0.13) ×10^7^ M^-1^ [HEPES/7.4/25]	40
TM	Neurotoxicity	CB^7^	Accelerate recovery from anaesthesia	(8.0±0.5)×10^4^ M^-1^ [E3/7.2/25]	59
MPTP	Neurotoxicity	CB^7^	Inhibit neurodegeneration	(4.8±0.2)×10^4^ M^-1^ [10 mM PBS/7.4/25]	60
MPP^+^	Neurotoxicity	CB^7^	Inhibit neurodegeneration	(1.05±0.05)×10^5^ M^-1^ [10 mM PBS/7.4/25]	60
PTZ	Neurotoxicity	CB^7^	Inhibit PTZ induced seizure	(1.94±0.11)×10^5^ M^-1^ [H_2_O/N.A./25]	61
CFZ	Cardiotoxicity	CB^7^	Decrease cardiotoxicity	10^4^~10^5^ M^-1^ [HCl-H_2_O/2.0/25]	62
BDQ	Cardiotoxicity	CB^7^	Decrease cardiotoxicity	3.98×10^3^ M^-1^ [CH_3_CN/Neutral/25]	63
SO	Cardiotoxicity	CB^7^	Decrease cardiotoxicity	(2.87±0.13)×10^5^ M^-1^ [D_2_O/7/25]	64
DPI	Cardiotoxicity	CB^7^	Decrease cardiotoxicity	(3.13±0.16)×10^4^ M^-1^ [H_2_O/Neutral/25]	65
		CB^8^	Decrease cardiotoxicity	2.26×10^12^ M^-2^ [H_2_O/Neutral/25]	65
AH	Hepatotoxicity	CB^7^	Alleviate hepatotoxicity	(1.21±0.12)×10^3^ M^-1^ [H_2_O/Neutral/25]	66
NC	Hepatotoxicity	CB^7^	Alleviate hepatotoxicity	N.A.	67
TZ	Hepatotoxicity	CB^7^	Alleviate hepatotoxicity	(1.50±0.13)×10^6^ M^-1^ [H_2_O/Neutral/25]	68
mCPP	Hepatotoxicity	CB^7^	Alleviate hepatotoxicity	(6.90±0.49)×10^5^ M^-1^ [H_2_O/2.4/25]	68
CPT	Cytotoxicity	CB^7^	Alleviate non-specific toxicity	9.5×10^7^ M^-2^ [DMSO-H_2_O/Neutral/N.A.]	69
OxPt	Cytotoxicity	CB^7^	Reduce cytotoxicity	2.89×10^6^ M^-1^ [20 mM PBS/6.0/37]	70
		WPA^6^	Reduce cytotoxicity	1.66×10^4^ M^-1^ [20 mM PBS/7.4/37]	88
PEI	Cytotoxicity	CB^7^	Decrease cytotoxicity	(1.03±0.19)×10^5^ M^-1^ per repeating unit [H_2_O/Neutral/25]	71
HB	Blood coagulation	CB^7^	Decrease coagulation effects	(1.04±0.19)×10^7^ M^-1^ per repeating unit [H_2_O/7.4/25]	72
NTX	Non-selective teratogenic toxicity	CB^7^	Alleviate teratogenicity	(1.4±0.15)×10^5^ M^-1^ [H_2_O/Neutral/25]	73
THI	Non-selective teratogenic toxicity	CB^7^	Alleviate teratogenicity	(7.46±0.10)×10^5^ M^-1^ [H_2_O/Neutral/25]	73
Cisatracurium	Neurotoxicity	CLBD1	Accelerate recovery from anaesthesia	(9.7±0.8)×10^5^ M^-1^ [N.A./N.A./N.A.]	79
Methamphetamine	Illicit drug	CLBD2	Reverse the hyperlocomotive activity	(4.3±1.0)×10^6^ M^-1^ [20 mM PBS/7.4/25]	81
Sch	Neurotoxicity	WPA^6^	Reduce side-effects	3.42×10^6^ M^-1^ [PBS/7.4/25]	90

Note: N.A. “not available”. PBS: phosphate buffer saline. R.T.: room temperature. HEPES: 4-(2-hydroxyethyl)piperazine-1-ethanesulfonic acid buffer.

## Abbreviations

CD: cyclodextrin
NMBA: neuromuscular blocking agent
nAChR: nicotinic acetylcholine receptor
ACh: acetylcholine
FDA: Food and Drug Administration
BA: bile acid
CA: cholic acid
DCA: deoxycholic acid
GCA: glycocholic acid
TCA: taurocholic acid
L/D-Tyr-β-CD: L/D-tyrosine-modified β-CD
L/D-Trp-β-CD: L/D-tryptophan-modified β-CD
Trm-β-CD: tyramine-modified β-CD
C[*n*]A: calix[*n*]arene
SC[*n*]A: *p*-sulfonatocalix[*n*]arene
PQ: paraquat
DQ: diquat
STC[4]A: *p*-sulfonatothiacalix[4]arene
UFH: unfractionated heparin
GC[4]AOEG: oligoethylene glycol functionalized guanidinocalixarene
CB[*n*]: cucurbit[*n*]uril
AD: amantadine
TM: tricaine mesylate
MPTP: *N*-methyl-4-phenyl-1,2,3,6-tetrahydropyridine
MPP^+^: *N*-methyl-4-phenylpyridine
PTZ: pentylenetetrazol
CFZ: clofazimine
BDQ: Bedaquiline
SO: sorafenib
SMKI: small-molecule kinase inhibitor
DPI: Diphenyleneiodonium
AH: arecoline
NC: nitidine chloride
TZ: Trazodone
mCPP: *m*-chlorophenylpiperazine
CPT: camptothecin
OxPt: oxaliplatin
PEI: polyethylenimine
HB: hexadimethrine bromide
NTX: nereistoxin
THI: thiocyclam
Acyclic CB[*n*]: acyclic cucurbit[*n*]uril-type
CLBD1: calabadion 1
CLBD2: calabadion 2
PA[*n*]: pillar[*n*]arene
WPA[*n*]: water soluble pillar[*n*]arene
Sch: succinylcholine
SPM: spermine
SPD: spermidine
PUT: putrescine
PEG: polyethylene glycol
P1PA5: peptide-PA^5^ conjugate
HP-β-CD: 2-Hydroxypropyl-β-cyclodextrin
β-CDP: β-CD based polymer
NPC1: Niemann-Pick type C1
NPC2: Niemann-Pick type C2
NPC: Niemann-Pick disease type C
